# A hybrid computational strategy to address WGS variant analysis in >5000 samples

**DOI:** 10.1186/s12859-016-1211-6

**Published:** 2016-09-10

**Authors:** Zhuoyi Huang, Navin Rustagi, Narayanan Veeraraghavan, Andrew Carroll, Richard Gibbs, Eric Boerwinkle, Manjunath Gorentla Venkata, Fuli Yu

**Affiliations:** 1 Human Genome Sequencing Center, Baylor College of Medicine, Houston, TX USA; 2 DNAnexus, Mountain View, CA USA; 3 Human Genetics Center, University of Texas Health Science Center, Houston, TX USA; 4 Oak Ridge National Laboratory, Oak Ridge, TN USA

**Keywords:** WGS, SNV, Variant calling, Joint calling, Supercomputer, Cloud AWS, Scalable, Big data, Ensemble calling

## Abstract

**Background:**

The decreasing costs of sequencing are driving the need for cost effective and real time variant calling of whole genome sequencing data. The scale of these projects are far beyond the capacity of typical computing resources available with most research labs. Other infrastructures like the cloud AWS environment and supercomputers also have limitations due to which large scale joint variant calling becomes infeasible, and infrastructure specific variant calling strategies either fail to scale up to large datasets or abandon joint calling strategies.

**Results:**

We present a high throughput framework including multiple variant callers for single nucleotide variant (SNV) calling, which leverages hybrid computing infrastructure consisting of cloud AWS, supercomputers and local high performance computing infrastructures. We present a novel binning approach for large scale joint variant calling and imputation which can scale up to over 10,000 samples while producing SNV callsets with high sensitivity and specificity. As a proof of principle, we present results of analysis on Cohorts for Heart And Aging Research in Genomic Epidemiology (CHARGE) WGS freeze 3 dataset in which joint calling, imputation and phasing of over 5300 whole genome samples was produced in under 6 weeks using four state-of-the-art callers. The callers used were SNPTools, GATK-HaplotypeCaller, GATK-UnifiedGenotyper and GotCloud. We used Amazon AWS, a 4000-core in-house cluster at Baylor College of Medicine, IBM power PC Blue BioU at Rice and Rhea at Oak Ridge National Laboratory (ORNL) for the computation. AWS was used for joint calling of 180 TB of BAM files, and ORNL and Rice supercomputers were used for the imputation and phasing step. All other steps were carried out on the local compute cluster. The entire operation used 5.2 million core hours and only transferred a total of 6 TB of data across the platforms.

**Conclusions:**

Even with increasing sizes of whole genome datasets, ensemble joint calling of SNVs for low coverage data can be accomplished in a scalable, cost effective and fast manner by using heterogeneous computing platforms without compromising on the quality of variants.

**Electronic supplementary material:**

The online version of this article (doi:10.1186/s12859-016-1211-6) contains supplementary material, which is available to authorized users.

## Background

Large cohort studies are extremely useful for discovering genotype phenotype associations and to characterize variation with great public health significance [1–4]. The decreasing costs of sequencing are increasingly making it possible to sequence whole genomes in the millions in the coming years [5]. The past decade has also seen the development of many joint calling approaches for genomic data produced with low coverage whole genome sequencing [6–8]. Joint calling is necessary for low to medium coverage sequencing projects (~10×) as it further reduces false positives rate especially at the rarer end of the site frequency spectrum. It is also clear that improving the yield of variants from sequenced data across the whole spectrum of variants requires the deployment of diverse statistical and algorithmic approaches [9–11]. It is also important to correct for algorithmic biases to ensure high fidelity variants [12]. Consensus strategies on ensemble calling of low coverage sequencing data in the 1000Genomes project [1] has produced variants with high sensitivity and low false discovery rate (FDR). Imputation strategies have also been shown to improve the variant discovery power of variant calling pipelines analyzing low coverage data [11, 13]. For example, the 1000Genomes Phase 3 (1000GP3) Project used multiple joint callers for site discovery, followed by a genotype likelihood step and an imputation and phasing step [1] for ~2500 whole genome low coverage samples. Given the projected growth in sequenced data in the coming years, variant calling pipelines will have to adapt to a computational footprint of an unprecedented scale and make it tractable both in terms of time and costs. It will require an extremely generous data storage facility and a massive number of cores. Variant calling of ~ 2500 whole genome samples in the 1000GP3 project took multiple institutions to collaborate over months to produce the final results. Huang et al. [14] estimate 1–2 months of exclusive access on a typical Local High Performance Compute Cluster (LHPC) to accomplish Single Nucleotide Variant (SNV) calling using SNPTools [8] for the 1000GP3 dataset.

The advent of cloud computing framework [15] has significantly boosted the ability to tackle problems of scale, with several existing cloud based solutions to process genomic data [16–21]. There has been some past work on porting state-of-the-art variant calling pipelines [22] for targeted whole exome sequencing of thousands of samples to the Amazon Web Services (AWS) [15] cloud [19, 21], but a cloud based ensemble calling workflow for thousands of whole genomes is lacking. Instance limits on data storage is a serious limitation for joint calling of large cohorts in the AWS environment, which is typically not a problem in an LHPC environment with sufficient capacity. Scaling up the LHPC infrastructure to meet the computational needs can prove to be costly as the cost of maintaining just a 100 node cluster can run up to 100,000$ a month [23, 24]. Large supercomputers typically deployed in computing centers and Department of Energy leadership computing facilities provide systems with large number of computing cores with specialized integer and floating point arithmetic, memory capacity, low-latency and high-bandwidth network, and high-capacity IO [25, 26]. However, most of these systems limit the execution time of a job to a few tens of hours. This is a major limitation to workloads such as joint calling of a large cohort of WGS samples, whose jobs are typically hundreds of hours [24].

In this work, we show that variant calling pipelines using a hybrid computational environment can leverage the strengths of each architecture to process cohorts with thousands of whole genome samples in real-time while minimizing operational costs. As a proof of principle, we present performance metrics of SNV calling on the Cohorts for Heart and Aging Research in Genomic Epidemiology WGS freeze 3 dataset (CHARGES-F3) [4] using three different computational environments. There are 5297 whole genome sequenced (WGS) samples in this dataset sequenced with 6 × –10× coverage for a total of 180 TB of aligned BAM file data. The variant calling workflow is divided into four stages, where **Stage A** is defined as the variant site identification stage involving four callers, **Stage B** as the consensus site filtering step, **Stage C** as the genotype likelihood step and **Stage D** as the imputation and phasing step. For the CHARGES-F3 dataset, Stage A, Stage B, Stage C and Stage D were completed on AWS [10], LHPC at Baylor College of Medicine, AWS and the large supercomputers at ORNL [20] and Rice [21] respectively. The four joint callers used in Stage A are SNPTools [8], GATK HaplotypeCaller (GATK-HC) [6], GATK UnifiedGenotyper (GATK-UG) and GotCloud [7]. The SNPTools genotype likelihood module and imputation and phasing module are used for Stage C and D respectively. We developed a tool called **g**enomic **S**ingle **N**ucleotide **A**nalysis **P**ipeline (goSNAP) for this project.

There were approximately 72 million SNVs called in CHARGES-F3 dataset, with approximately 50 and 60 % novel with respect to 1000GP3 and dbSNP141 databases respectively. Using a strategy which includes all sites which have been called by at least 3 callers (consensus 3of4), we ensured false discovery rate (FDR) < 3.34 % and specificity of over 99 % in the final callset with respect to a golden dataset consisting of whole exome sequenced samples with 80–100× coverage. The entire operation was finished in 50 days with a total core hour usage of ~ 5.2 million across all the infrastructures (see Table 2). Each aligned BAM file was split into 1 Mbp region for joint calling on AWS. This created a cache data footprint of 360 TB with a time to live not exceeding 14 days. Only 6 TB of data was transferred across all platforms. The goSNAP pipeline is designed to minimize egress charges, data storage charges and data transfer costs. It optimizes on concurrent core usage to be cost effective and fast. To the best of our knowledge, ensemble calling on a WGS cohort with over 5000 samples has not been done before and this approach can be easily scaled to 10,000 samples (see Discussion).

## Results

The workflow for the goSNAP pipeline has been designed to address the scalability challenges in large scale genomic computing, and to minimize egress charges and computational time, while ensuring high quality results in variant calling. When scaling up, the major computational bottlenecks are the joint calling and imputation and phasing step. To address these challenges, we have designed and tested a hybrid computational paradigm, which consisted of (1) a Local High Performance Cluster (LHPC) made up of commodity hardware; (2) Amazon Web Service (AWS) [15]; and (3) and the supercomputers at ORNL (e.g. Titan, Rhea) [25] and at Rice University (Blue BioU) [26]. In this study, we demonstrated the feasibility of using a hybrid computational paradigm in processing large-scale genomic datasets by applying this to the CHARGE WGS data consisting of 5297 samples (Methods and Additional file 1).

### Challenges in scalabilities for large-scale genomic data processing

#### Limitations

**Fig. 1:**
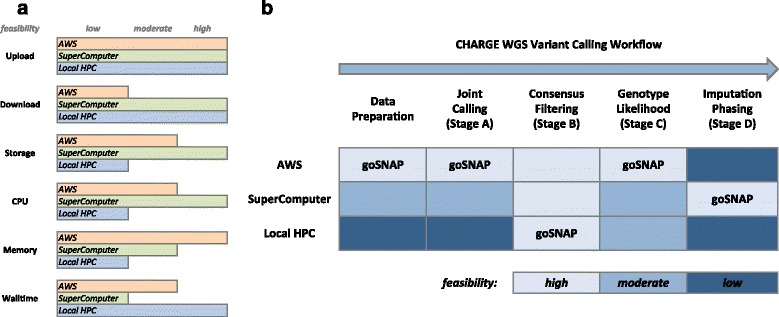
**a** A resource constraint analysis diagram of computing resources with respect to the three available architectures. In this diagram feasibility is measured in terms of cost, time and resource bounds. **b** A resource constraint analysis diagram of the variant calling stages for CHARGES-F3 dataset. Feasibility is measured in terms of cost, time and limited computing infrastructure in the AWS cloud environment, Supercomputer and LHPC respectively. The measure of feasibility is for illustration purposes only and does not conform to any data

Most LHPCs with typical research environments have few PBs of storage and millions of core-hours per month and are constrained by hardware limits on data storage, computing power and data transfer bandwidth (see Fig. 1a) to carry out large computes. Scalability is not a problem for the AWS computing environment as it allows flexibility to increases the compute and data resources with a ‘pay per use’ model [27]. However, the outbound data transfers incurs a cost which scales linearly with the amount of data transferred (see Fig. 1a). It is also necessary to optimize on all aspects of the compute including memory bandwidth and capacity (RAM), computing cores (CPU) and IO capacity and bandwidth (HDD) to make optimal use of the instances and achieve cost-effectiveness. For projects involving big data, there is an additional cost of implementing data parallelization to overcome the limitations of local instance on HDD space. The large supercomputing infrastructure has an extremely large data store, premium hardware optimized for high IO bandwidth, low-latency and high bandwidth network, and dedicated hardware and software support for CPU-intensive operations, but computing jobs have to finish within hard wall time limits. For example, Titan at ORNL [25] requires all jobs to finish within 24 hrs. Scheduling delays in allocating large number of resources can add to the turnaround times.

#### Challenges

Executing the entire pipeline in Fig. 1b on any single platform can be challenging for many reasons. The storage and compute requirements of Stage A are beyond the capacity of most LHPCs. Implementing Stage A in the AWS environment requires a workflow which minimizes egress charges apart from splitting and replication of data for joint calling to contend with limited per instance HDD space. Doing Stage A on supercomputers is not scalable as memory intensive variant calling jobs get in the way of achieving high concurrency on uniform hardware (see Fig. 1b). For example, our profiling suggests that GATK-UG needs approximately 16GB of RAM per joint calling job across 5000 samples. On a supercomputer like Rhea [25] with 256 nodes, and 64GB memory per node, no more than 1000 jobs can be scheduled concurrently. Unanticipated batch failures in the presence of maintenance downtimes and fair share scheduling policy can also adversely affect turnaround times for the whole project. Variation in coverage can cause job failures across all the infrastructures.

Stage D does not require IO, RAM or HDD space, but LHPC resources are still inadequate for Stage D. Using the AWS environment for Stage D will be an inefficient utilization of the instances as they are billed for the entire configuration and not just processing power. In our profiling step, best practices configuration for SNPTools imputation module failed to finish within the maximum 24 hrs wall time limit on Titan [25]. While Blue BioU [26] was successful in finishing the imputation and phasing step within 24 hrs on a sample bin, it did not have sufficient capacity to ensure timely completion of the entire Stage D computation.

#### Solution

**Fig. 2:**
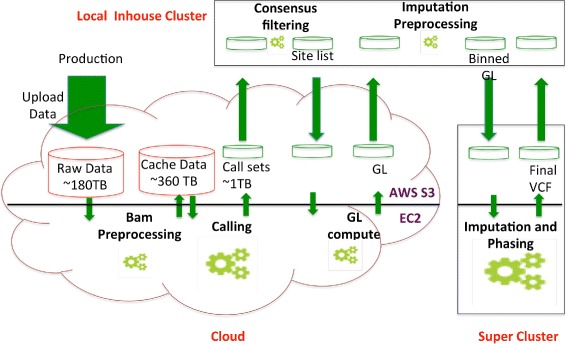
goSNAP pipeline workflow minimizes egress charges. Variant calling (Stage A) and genotype likelihood (Stage C) calling is done on the AWS cloud, consensus filtering and imputation preprocessing is accomplished in the LHPC (Stage B) and Imputation and Phasing (Stage D) is done at the supercomputers at Rice and Oakridge National labs

After extensive profiling and analyzing all the pros and cons of the three infrastructures, the goSNAP pipeline ported Stage A on AWS, Stage B on LHPC, Stage C on AWS and Stage D on Rhea and Blue BioU (see Fig. 2). In the rest of this section, we first present SNV calling results and then present performance metrics of the goSNAP pipeline on the hybrid computational environment.

### Results of SNV calling on 5297 low coverage whole genomes

In Table 1, the results for joint calling of 5297 low coverage whole genomes is presented. All sites which have been called by at least three callers (consensus 3of4) are included for further analysis. There are approximately 72.9 million bi-allelic SNVs which have been called using consensus 3of4 approach from the four variant callers GATK-HC, GATK-UG, SNPTools, and GotCloud. The transition-to-transversion ratio (Ti/Tv) of 2.12 is consistent with past results [1]. While consensus 2of4 strategy gave ~86 millions SNVs, the FDR is 11.29 % with respect to WES gold standard dataset (see Table 1). The consensus 3of4 callset only has a FDR of 3.34 % with the same gold standard dataset. The sensitivity is greater than 95 % for allele count >5 (see in Additional file 1: Figure S2), whereas the sensitivity is ~43.1 and ~72.2 % for singletons and doubletons respectively. The consensus 3of4 callset has higher specificity and lowest FDR when compared to  all the callers. The number of SNVs recovered in 1000GP3 and dbSNP141 is also the highest among all the callers. All callers have unique variants in their callset (see in Additional file 1: Figure S1), with GotCloud having the lowest number (~580,000) of unique variants with respect to the CHARGE-F3 cSNP dataset. The genotype concordance is 98.7 % for (Ref/Ref), 84.1 % for (Ref/Alt) and 99.3 % for (Alt/Alt) when compared to CHARGE cSNP array gold standard dataset.

**Table 1:** Variant calling sensitivity and specificity for the consensus 3of4 approach ensures high specificity and FDR without a loss of sensitivity

	Consensus 3of4	Consensus 2of4	GATK-HC	GATK-UG	GotCloud	SNPTools
# SNVs	72,945,834	86,233,412	103,439,411	104,649,069	78,483,824	66,290,585
Ti/Tv	2.12	2.08	2.00	2.00	2.09	1.99
% in 1000G	50.22 %	43.75 %	36.35 %	36.54 %	46.91 %	51.17 %
% in dbSNP	40.25 %	35.31 %	28.88 %	29.53 %	37.93 %	41.91 %
sensitivity	63.80 %	68.98 %	68.51 %	69.99 %	64.17 %	51.26 %
specificity	99.92 %	99.70 %	99.30 %	99.54 %	99.86 %	99.13 %
FDR	3.34 %	11.29 %	22.91 %	16.16 %	6.12 %	33.11 %

The gold standard dataset consists of 4612 samples with 80–100 × coverage. All the four callers are necessary for increasing the yield of SNVs

### Application of our hybrid computational paradigm to variant calling of 5297 WGS dataset

In our computational model, 5297 aligned WGS BAM files (~180 TB) are uploaded to the AWS environment. In the slicing and repacking stage, we slice the BAM of each sample into windows of size 1 Mbp and repack the sliced BAM from all samples in the same window into the one data package (see Method) for joint calling. This size is empirically determined (see Methods) to fit into AWS instances with HDD space not exceeding 320GBs for the joint calling jobs, as well as to reduce the number of intermediate files, which improves the efficiency of data access and transfer between EC2 and S3. Both the slicing and repacking jobs use “xargs” parallelization to make full use of the instance CPU cores, which ensure that none of the cores remain idle and improves the runtime by up to 8 fold whenever possible. Several configurations were tested in the xargs mode ranging from 1 to 32 cores for the slicing stage, and the most cost effective instance had 8 cores, 16GB RAM and 160GB solid state HDD space (see Table 2). The repacking stage predominantly uses instances with 1660GB of HDD space to accommodate the data across all the samples (see Methods). The slicing and repacking of 200 TB whole genome BAMs takes 2 and 3 days, respectively (74,000 core hours). During this process, the data is temporarily duplicated twice, and the cache data copy is removed after the joint calling.

**Table 2:** Summary performance metrics of goSNAP pipeline

Stage	# of core hours	Time (days)	Data (TB) generated	Data (TB) (upload/ download)	Median execution time /unit	# of parallel execution threads	Optimal instance (core/mem)
Slicing /Repack	~48 k/~26 k	5	360	180/0	~1.15 h/sample (slicing)/~22 h/bin (repacking)	5297 (slicing) 300 (repacking)	8 cores, 16GB/ 4 cores, 4GB
Calling	~1.4 million	14	120	0/2	~60 hrs/bin	2797	8 cores, 16GB
Genotype Likelihood	~15.6 k	1	6	0/2	~1.5 h/BAM	5297	2 cores, 8GB
Imputation and Phasing	~3.7 million	30	2	2/2	~30 h/bin @ Rhea ~13 h/bin @ Blue BioU	265 k	32 cores
Total	~5.2 million	50	488	182/6	--	--	--

The pipeline finished in 50 days and only transferred a total of 6 TB of data starting from a raw data footprint of 180 TB. Cache data of 360 TB was live only for 14 days. Intermediate results amount to 120 and are archived for future use

The pipeline used 5.2 million core hours

In the joint calling stage, the four variant callers, namely GATK-HC, GATK-UG, SNPTools and GotCloud are grouped into one AWS compute job to call variants in each 1 Mbp region. Each caller is employed in joint calling mode, and any caller specific per sample processing, for instance gVCF calling in GATK-HC and effective base depth (EBD) calculation in SNPTools, is fully parallelized, which effectively reduces the runtime of the joint calling step by approximately 5-folds. The joint calling of 5297 genomes only took 2 weeks (1.4 million core hours), with an average 60 hrs per 1 Mbp region.

The average concurrency is 8000 cores per hour, with the peak concurrency 10,000 cores per hour. Since any failure that occurs during the long-running instance jobs is very costly, we run all the tools in the failure checking and retry mode (see Method). The average proportion of runtime taken by GATK-UG, GATK-HC, GotCloud and SNPTools is 40 %, 30 %, 20 % and 10 % respectively. Nine types of instances (in Additional file 1: Table S1) are used in the joint calling stage, of which 56 % of the jobs use an instance with 8 cores, 61GB RAM and 160GB HDD space. 96 % of the jobs used instances less than 320GB of HDD space, with four configurations each using at least 18 % of the total number of jobs. These instances are cores = 8, RAM = 61GB, HDD = 160GB (34 %); cores = 16, RAM = 122GB (22.5 %), HDD =320GB; cores = 8, RAM = 15GB, HDD = 160GB (21.5 %); cores = 16, RAM = 30GB, HDD = 320GB (18 %). The remaining instances were used to accommodate the variation of sliced BAM size due to depth variation across the whole genome. To reduce the egress charge in the cloud, we split the joint calling results into two parts, the essential data, containing the multi-sample VCFs from all callers, and the auxiliary data, containing intermediate files, like per sample gVCFs or EBD files. The total amount of output from the whole genome joint calling is 120 TB, of which only 2 TB of essential data are downloaded to LHPC for site level consensus filtering and quality control purpose. All auxiliary data are archived for future research project.

The site level consensus and quality control is performed on the LHPCs, and a union variant site list is generated, which is uploaded back to AWS for genotype likelihood calculations (GL) using SNPTools (see Fig. 2). The computational resources required for the GL step is not challenging for most of the infrastructures, but since the input whole genome BAM files are stored in the cloud, doing this stage (Stage C) on the cloud prevents egress charges. The per sample genotype likelihood data is only 2 TB. It is downloaded to Rhea and Blue BioU for imputation and phasing. Compared to 200 TB alignment data, 120 TB variant calling data, only 4 TB calling and GL data is downloaded and charged (see Table 1).

Imputation and phasing is the most compute intensive stage. We take the GL data as input and run the imputation and phasing on Rhea and Blue BioU using SNPTools imputation engine (see Methods). The optimal imputation window size is decided taking into account the population diversity and the imputation runtime without exceeding the wall time limit of the supercomputer. The imputation jobs are scheduled according to the specification of each compute node, in order to make full use of computing resources and reduce the job scheduling overhead. The reference independent imputation of 5297 samples took 4 weeks, with 3.7 million core hours (see Table 1) including the system maintenance downtime and scheduling delays due to fair-share policy. The average runtime of imputing a bin with 512 SNVs is approximately 13 hrs on Blue BioU and 30 hrs on Rhea.

## Discussion

Ensemble joint calling of 5297 WGS samples is an unprecedented undertaking to the best of our knowledge. The limitations of joint calling tools for a sample size of this scale  had not been tested for, and a successful completion of the whole compute requires all protocols to be robust to resource allocation failures and silent faults [28]. Non uniform coverage of the samples can contribute to unanticipated failures and data replication costs can adversely affect the operational costs. For example, five bins with large sizes were removed from further analysis in the goSNAP pipeline, because the jobs did not finish even after 120 hrs of runtime. The entire pipeline only replicated the data in Stage A (~360 TB) for 14 days and it is easy to reduce the cache data size to less than 200 TB by using a strategy where sliced bins are deleted as soon as the repacking stage finishes on the sliced bins. Using multiple callers can add to the challenges due to scale, but are necessary to ensure higher sensitivity as all the callers used in our pipeline have distinct algorithmic strategies. In the goSNAP pipeline all the callers contributed unique variants at the end of Stage A when compared to the highly polymorphic cSNP array (see in Additional file 1: Figure S1). Three callers have higher sensitivity compared to consensus 3of4 approach but the smallest FDR value (GotCloud) among the callers is ~6 % which is almost twice that of the consensus 3of4 approach (see Table 1). SNPTools has lower sensitivity than consensus 3of4 but recovers most common variants (1000G, dbSNP) compared to the other callers at only 10 % of the computation resource cost. Using some combination of three callers may only improve the sensitivity by a maximum of 7 % but the FDR might also be very high, as the FDR statistics for the consensus 2of 4 approach indicate. Suppose we add one more caller and use 4 of 5 consensus strategy, the sensitivity may at best reach the sensitivity of GATK-UG (69.99 %), a maximum gain of 7 % compared with the sensitivity of 3 of 4 consensus (63.80 %) as in Table 1, but the FDR of 4 of 5 is not likely to be much lower than that of 3 of 4 consensus (3.34 %), compared to the FDRs of all four callers currently employed. On the other hand, the computation cost scales at best linearly with number of callers used. Therefore the strategy of using four callers for variant site identification followed by consensus filtering was necessary for our project to ensure high sensitivity while maintaining low FDR statistics. In general, the decision to include more callers should depend on a number of factors such as, number of samples, depth of coverage, computing budget and project deadlines.

The yield of novel variants compared to 1000GP3 and dbSNP are also likely to be of a high quality because the Ti/Tv ratio of 2.12 is consistent with past results [1]. Sequencing errors can lead to a decreased Ti/Tv ratio due to introduction of random noise, especially at the rarer end of the site frequency spectrum. All the four callers in our pipeline have a Ti/Tv ratio less than the consensus 3of4 approach (see Table 1) and higher FDR statistics, thereby indicating that consensus filtering strategy reduces random noise. The site level consensus filtering minimizes the need of any tool specific filters (e.g. VQSR in GATK and SVM in GotCloud) as the FDR of the consensus 3of4 approach is at least half as low as any individual caller (GotCloud). While the overall sensitivity is only 63.80 %, the sensitivity is over 95 % for allele frequency > 0.001 (see in Additional File 1: Figure S2). This behavior is consistent with past work in detecting singletons and doubletons from low coverage data [20, 29]. The yield of SNVs from our pipeline is less than that of 1000GP3 (~84 million) [1] even though the number of samples is almost twice as large, but that can be attributed to the relatively homogeneous ancestry of our samples compared with 1000GP3. In a previous paper [30] on ~1000 samples from our dataset, the yield of SNVs was ~ 24 million, which is less than that of a comparable sample size of 1000Genomes Phase 1 project. Even the UK10K SNV callset [2] has only ~42 million SNVs whereas the number of unrelated samples in that project exceeds that of 1000GP3.

A cloud based joint calling framework has been discussed in Shringarpure et al. [20] where a single joint caller is used to call 1000GP3 dataset. In their work, samples for the same population are grouped together for joint calling and calling is done one chromosome at a time. This strategy may fail with increasing sample sizes even with the most powerful instances. For example, the CHARGES-F3 dataset has been sequenced with higher per sample coverage than 1000GP3 dataset and the sample size of EuAm (~3700) alone exceeds that of 1000GP3 (~2500). The data footprint of Chr22 in the CHARGES-F3 dataset is 4 TB and of Chr1 is 18 TB which can only be accommodated on the D2 Dense storage instances in AWS [31]. Our binning strategy provides a more scalable alternative, as 96 % of the nodes used in our work used HDD space less than 320GBs and can scale easily to much larger sample sizes. We did not face any scheduling delays which could be an issue when using high memory nodes on AWS instances [32]. In our binning strategy, all mapped read mates, unmapped read mates and 10 Kbp mapped reads in the buffer region are included in the sliced 1 Mbp bin for joint calling. Since many indel callers like GATK-HC and GATK-UG use this information to call high quality indels, our binning strategy minimizes errors in indel calling especially near the ends of the bins.

Joint calling approaches on typical LHPCs and supercomputing infrastructures has also been studied in the past. In [27], the authors use a supercomputing platform with ~16,000 cores and 72 hrs wall time to do joint calling using GATK-HC on 437 whole genomes with an average of ~30× coverage. They estimate a quadratic increase in resource usage consumption as sample size increases, and to make it feasible on their supercomputing platform, they perform group variant calling on subsets of their entire cohort in an ancestry dependent fashion. However, comparing the effective number of reads per ethnicity group in their work (less than 400 samples each with 30× reads), with the one in our dataset, about 3000 samples in a single population with 8× average coverage, our effective number of reads in the joint calling is at least doubled. This renders it less feasible to perform per population joint calling with our sample size scale on a supercomputing platform, as the resource consumption scales quadratically with the sample size. In the paper [27], the authors also include performance analysis of aligning raw read data as part of their computational footprint. In our workflow, we assume that the raw sequencing data is aligned and uploaded to AWS as it is sequenced, as storing raw sequencing data of over 5000 samples (>200 TB) can be infeasible for an LHPC environment attached to a sequencing facility. Aligning and uploading to AWS in batches also minimizes data transfer bottlenecks across computing infrastructures and can be readily tuned to match sequencing throughput. The rate limiting step in the alignment and sequencing stage is likely to be sequencing throughput, as the expected time to sequence ~5000 whole genome samples with the current sequencing capacity far exceeds the turn around time of the entire goSNAP pipeline. Since the alignment is usually performed at the per sample level and the computation time scales linearly with the sequencing depth, we consider sequencing and alignment as a single stage with well controlled turnaround time. Hence the alignment can be performed gradually as the sequencing reads are available, on a local cluster at the sequencing center, and the aligned data can be uploaded to the cloud for joint calling, where the computational resources are more abundant.

In the context of variant calling, the advantages of using an LHPC environment do not supersede the remaining two resources (Fig. 1b) for any stage and an argument can be made to design future variant calling pipelines which are either solely based on an AWS environment or on a large supercomputer. However, in this project, all the real time QAQC and job tracking was carried out on an LHPC environment. Despite the limited resources on an LHPC, the flexible computing environment aids in the rapid development of QAQC tools which in turn mitigates risk by ensuring the integrity of the goSNAP pipeline at the start and at the end of each stage. Several current tools for genomic data have been already designed for an LHPC environment but have to be ported for use in an AWS or supercomputing environment, thereby adding to the timelines of a large project like CHARGE. Even though we did not use LHPC for variant calling on any bin in the CHARGE dataset, a scenario can be anticipated where variant calling on some regions of the genome may only be feasible on an LHPC, with execution parameters different than the rest of the pipeline. Changing the execution parameters on some selected bins may make it infeasible to execute on either the AWS or supercomputing environment without additional development and testing of software.

There are several challenges in scaling up the analysis to even larger samples sizes. In the joint calling stage, increasing sample sizes will require instances with much larger instance HDD space. In the current implementation, the sliced per region per sample BAM files are grouped together using tar compression. This helps to significantly reduce the number of input files. But the downside of tar compression is that it requires almost double the HDD space for the decompression. To work around with the instance storage limitation and to cope with the upcoming larger sample size, we propose an in-place compress-decompression strategy, by binary concatenating small tarballs together for input/output data transfer, and binary truncating the data chunks before decompression. The turnaround decompression space is expected to be as large as the small tarball size. This is an important direction for the future work. However, even with the current binning size parameters, increasing the sample size to 10,000 samples would increase the size of the bins to only 120GB for 80 % of the bins. This can be scheduled with the existing goSNAP release version, as 96 % of the instances used in this run had 320GBs of HDD space and 60 % of instances used less than 62GB of RAM. It can be projected that with 10,000 samples, the RAM requirements will double at most, and the configuration with 122GBs of RAM and 320GBs of HDD space will suffice for 60 % of the bins. Implementing the new strategy with trunc can further alleviate HDD space constraints. Furthermore, a strategy involving a smaller bin size can be used to mitigate the adverse effects due to using costly instances when scaling up to large sample sizes. For example, in our simulations we observe a 1.5× increase in run time on 100 Kbp bins with respect to 1 Mbp bins, with each bin taking approximately 9 hrs on each 100 Kbp region for approximately 3000 samples (see Methods). With 10,000 samples and a bin size of 100 Kbp this run time is projected to last for at most 40 hrs for most of the bins and twice the memory requirement of the current run. The number of jobs to manage will scale by 10 times for a sample size of 10,000 samples and bin size 100 Kbp but should not cause any performance degradation on the AWS system. The imputation and phasing module scales linearly with sample size and can finish easily within the 120 hrs wall time limit of Rhea. Reducing the bin size for imputation and phasing could be challenging for a diverse cohort, but the current bin size estimation will hold for a larger dataset with a homogeneous population. Though the number of burn-in iterations cannot be changed for larger sample sizes, the number of SNVs/bin can be optimized to fit into the wall time restrictions. Since all the data transferred in the goSNAP pipeline is variant information, increasing sample sizes will only increase egress charges proportional to the variant information in the samples.

Single sample calling for low coverage data will give a very high FDR [29], therefore joint calling is necessary for minimizing FDR and to improve sensitivity of variant discovery for low coverage datasets, even when the variant calling pipelines do not include Stage D. Even though deep coverage sequencing also does not have perfect recovery of singletons and doubletons [32], single sample calling gives comparable SNV callsets to multisample calling for high coverage datasets [29]. However, for calling indels and mnp variants, joint calling approaches may still be better than single sample calling. This is particularly relevant in the case of clinical sequencing, where increasing the sensitivity in calling indels may have prognostic significance.

The binning strategy can be used to scale up to sample sizes of 10,000 and beyond. However, to effectively use the computing infrastructures, the tools have to evolve to emerging architectures and data sizes. First, to efficiently use the supercomputers the tools have to be adapted for heterogeneous supercomputers. A significant number of highly scalable supercomputers, including Titan at ORNL are heterogeneous computers i.e., they use computing accelerators such as GPUs and Intel Many Integrated Core Architectures for achieving computation and power efficiency. Titan is the fastest supercomputer in the United States and it uses GPUs as computing accelerators. As this trend in supercomputer architecture continues [33], it becomes important for all tools to evolve to heterogeneous architectures. Second, as the data size increases with sample sizes, it is important to have data transfer protocols to exchange data among multiple points of computation that are geographically separate. Though our data transfer times in the current work were always less than 2 days, we anticipate the data transfer times becoming a bottleneck as we scale up to larger samples. Third, a smart job scheduler that can schedule jobs on hybrid computing infrastructure which includes Cloud, Supercomputers, and local computing infrastructure can decrease the burden on researchers to schedule and manage jobs.

## Conclusions

With increasing number of genomic datasets freely available on the AWS cloud [34], the next generation of variant calling pipelines will also be increasingly common in the AWS environment. While the costs of storage and compute cores in the AWS environment is declining, it may still be prohibitively costly to carry out many steps of standard variant calling workflow on the cloud. A hybrid computational approach involving multiple HPC systems may be an important future direction to explore. Our work on the goSNAP pipeline demonstrates that using a hybrid computation strategy can be cost effective and fast even with thousands of individual genomes.

## Methods

### Sequencing and alignment

There are 5297 WGS samples in the CHARGE-F3 dataset. They were sequenced using the ILLUMINA HiSeq 2000/2500 with an average depth of coverage ranging between 7× and 10×. The raw data was aligned using the Mercury pipeline [21]. The mercury pipeline used BWA to align the raw data to the human hg19 reference genome. The samples consist of three cohorts CHS [35], FHS [36] and ARIC [37] with 3396 samples belonging to European American (EuAm) ancestry and 1901 with African American (AfAm) ancestry.

### Golden datasets for comparisons

There are two golden datasets which are used in this paper. In Table 1 the golden dataset consists of 1782 and 2830 whole exome sequenced (WES) data from the AfAm and EuAm ancestry respectively. The WES dataset has been sequenced with 80–100× coverage. The WES gold standard dataset was aligned with the Mercury pipeline [21] in single sample mode. The second gold standard dataset which we refer to as cSNP, consists of 3533 samples genotyped with HumanExome BeadChip v1.0 (Illumina, Inc., San Diego, CA) querying 247,870 variable sites using standard protocols suggested by the manufacture at the University of Texas Health Science center at Houston [38]. There are 1683 EuAm samples and 1850 AfAm samples in the gold standard dataset. All true negative sites with missing genotype data are removed from the gold standard. For the sensitivity calculations, all sites common to WGS dataset with greater than 5 % missing genotypes are also removed from further analysis.

### Description of computing infrastructure

#### Local HPC at Baylor HGSC

The local compute cluster available at the Human Genome Sequencing center consists of 3800 cores with an average memory of 6GB per core. There is an aggregate of 4.5PB of live storage. There are no wall time limitations.

#### Rhea at ORNL

Rhea is a 512 nodes commodity type cluster located at the Oakridge Leadership Compute Facility [25]. Each node consists of two 8-core Intel Xeon processors which gives 32 concurrent threads of execution using hyper-threading. It is connected to 32PB Luster file system Atlas [25]. For the data processing of CHARGE project imputation, we had access to 256 nodes with a wall time limitation of 120 hrs.

#### DNAnexus AWS

DNAnexus is an automated AWS EC2 management platform providing a web based and command line interface to the AWS cloud infrastructure [39]. The specification of the EC2 instances used in the CHARGE processing is described in the Additional file 1. There is no wall time limit to the computing jobs. The backend data storage uses the AWS S3 service, with no storage limit but subject to storage cost.

### Deployment of goSNAP Pipeline

The slicing/repacking stage, the variant calling stage and genotype likelihood calculation (Stage A and C in Fig. 1b) were accomplished on AWS cloud system using the DNAnexus platform. The consensus site list was generated on the LHPC at BCM-HGSC with minimal egress costs (see Table 1). Imputation and phasing were accomplished on Rhea [25], an Oakridge leadership compute window is used as input of four variant callingFacility system and on Blue BioU [26], an IBM Power PC supercomputing facility at Rice University (see Fig. 2). All intermediate realtime QAQC was carried out on the LHPC at BCM-HGSC. This local compute facility was also used to monitor the jobs and to transfer data across infrastructures.

### Slicing and Repacking

In the slicing stage, the BAM file and index file of each sample is copied to an EC2 instance with 8 CPU cores, 16GB RAM and 160GB of hard disk storage (see Table 1). It is then sliced into 2897 BAMs, each with 1 Mbp region plus additional 10 Kbp of flanking regions which overlap with adjacent regions. We use “samtools view” to slice the BAM and “xargs” to parallelize the slicing jobs to make full use of all CPU cores. Every ten adjacent 1 Mbp BAMs and the index files are further compressed into a 10 Mbp tarball in parallel and transferred back to S3 data storage. The grouping of 10 regions reduces the count of intermediate files by over 50 k-folds between the slicing and repacking stage, which significantly improves the S3 bucket file access efficiency. At the end of slicing stage, we have three hundred 10 Mbp region directories, each containing 5297 per-sample tarballs of sliced BAMs and BAIs. The average size of a 10 Mbp tarball is 110MB per sample, which allows for an ECS instance with moderate disk storage to hold 5297 such tarballs for repacking. At the end of this stage, we have doubled the data footprint in S3.

In the repacking stage, 10 Mbp region tarballs of all 5297 samples are transferred from S3 into an ECS instance with 4 CPU cores, 4GB of memory and 800GB of disk space. The per sample 10 Mbp tarballs are decompressed and recompressed in parallel into 10 tarballs, each with BAMs and BAIs in the same 1 Mbp region from all samples. The parallelization is done using xargs. The repacked 1 Mbp BAM tarball with average size 60GB is stored in S3. Out of a total of 2897 regions, 163 one Mbp regions are empty as they intersect with centromere and telomere regions of the genome. The remaining 2734 one Mbp BAM tarballs are generated as input for joint variant calling.

### Choice of joint calling window size

We profiled the joint calling runtime of 10 random 1 Mbp regions and 10 random 100 Kbp regions using 4 types of EC2 instances. The minimum runtime is 9.83 hrs with 100 Kbp window size (8cores, 16GB RAM) and 60 hrs with 1 Mbp window size (8cores, 16GB RAM). The results of our profiling step for the goSNAP pipeline are presented in Additional file 1: Table S3.

Joint calling with 100 Kbp window size increases the overall runtime by approximately 50 %. For 1 Mbp regions, our profiling suggests that GATK-UG alone uses up approximately 40 % of the runtime of the entire goSNAP pipeline (see in Additional file 1: Table S4). The maximum window size of a region is limited by the instance storage. Since the average size of 1 Mbp input compressed BAM tarball is ~60GB, we use ECS instance with 160GB storage for the joint calling of most of the regions. Larger window size renders the process less scalable, as the high performance instance type with 500GB or larger, is either more costly on-demand or more scarce in spot instance mode. Therefore we take 1 Mbp as the optimal window size for joint calling for CHARGE Freeze 3 dataset.

### Joint calling

The repacked tarball of all sliced BAMs in the same 1 Mbp window is used as input of four variant calling pipelines, namely GATK-HC [6], GATK-UG [6], GotCloud [7] and SNPTools [8]. While SNPTools, GotCloud and GATK-HC are used to call variants in the whole 1 Mbp region, GATK-UG is called in a series of 10 windows of 100 Kbp size with maximum 20 retries in each window to prevent the job from quitting due to a known sporadic error [40]. A naive scheduling methodology based on tarball size is used to assign instances to bins as a first step (Additional file 1: Table S2). A complete list of type of instances used for the joint calling is given in Additional file 1: Table S1. The essential site level VCFs from each caller are downloaded to the LHPC for further consensus filtering, while the auxiliary variant calling results are archived using AWS. GATK-UG and GATK-HC also called indels as part of the pipeline.

### Choice of imputation window size

Imputation and phasing was profiled using SNPTools imputation engine over 3176 samples on Titan, Rhea, Blue BioU and LHPC. We profiled the imputation runtime of 1024 SNVs per window and 512 SNVs per window, both with 56 iterations, which is empirically determined to be sufficient for the SNPTools Markov Chain Monte Carlo (MCMC) algorithm to converge to a stationary distribution. While no simulation on Titan [25] could be finished due to wall time constraints, it took approximately 20 hrs, 58 hrs and 35 hrs on Blue BioU, Rhea and LHPC respectively, with 1024 SNVs per window, and 10 hrs, 24 hrs and 18 hrs on Blue BioU, Rhea and LHPC, respectively, with 512 SNVs per window. While 1024 SNVs/bin with 100 MCMC iterations is considered the best practices for SNPTools imputation for the 1000 Genomes samples with much higher population diversity, the relative homogeneity of the populations in the CHARGES-F3 samples allows for more relaxed requirements on the window size, with the same phasing accuracy. Therefore we choose 512 SNVs per window as the optimal imputation window size.

### Imputation and phasing

Imputation is accomplished using SNPTools [8] deployed on Rhea Supercomputer at ORNL [25] and Blue BioU high performance cluster at Rice University [26], the window size used for imputation and phasing is 512 SNVs per window with an overlap of 256 SNVs between adjacent windows. Across the autosome, there are a total of 285,000 imputation windows. The Monte Carlo Markov Chain step in each window was executed for 56 iterations on a single core with 55 burn-in iterations.

## Availability of supporting data

Project name: goSNAP

Project home page: http://sourceforge.net/projects/gosnap/

Archived version: 01d767

Operating system (s): Linux

Programming language: Python3, bash

Other requirements: Python3.2 or higher

License: Apache License v2.0

Any restrictions to use by non-academics: license needed

## Additional file

Additional file 1:
**Figure S1.** Venn Diagram of number of SNPs called by GATK-HC, GATK-UG, SNPTools and GotCloud and the FDR using HumanExome BeadChip array with 3533 shared samples as control. **Figure S2.** Rediscovery rate of SNP in the Exome region as function of alternate allele frequency using CHARGE WES variant call set as gold standard. The rediscovery rate exceeds 95% when alternate allele frequency f>=5x10^-4^ (AC>=5). **Table S1.** The choice of AWS instances used to deploy goSNAP for CHARGE WGS variant discovery in Stage A. All the jobs were scheduled via DNAnexus platform. Note that this list does not include the jobs for slicing and repacking in Stage A. **Table S2.** Instance specs in the “cost-effective” and “time-sensitive” mode of running goSNAP. **Table S3.** Profile of goSNAP runtime (in hour) with different region size, 100﻿ Kbp and 1 Mbp, and different instance specifications. **Table S4.** Profile of GATK-UG runtime (in hour) with different region size, 100 Kbp and 1 Mbp, and different instance specifications. (DOCX 232 kb)

## Abbreviations

1000GP3: 1000 genomes Phase 3
AfAm: African American
AWS: Amazon web services
BAM: Binary alignment/MAP
CHARGES-F3: Cohorts for heart and aging research in genomic epidemiology freeze 3
EuAm: European American
GATK-HC: GATK HaplotypeCaller
GATK-UG: GATK UnifiedGenotyper
GB: Gigabyte
goSNAP: Genomic single nucleotide analysis pipeline
HDD: Hard disk drive
Kbp: Kilo base pair
LHPC: Local high performance cluster
MB: Megabyte
Mbp: Mega base pair﻿
MCMC: Markov Chain Monte Carlo
PB: Petabyte
SNV: Single nucleotide variant
TB: Terabyte
Ti/Tv: Transition-to-transversion ratio
WES: Whole exome sequenced
WGS: Whole genome samples
