# Supplementary material for: A hybrid computational strategy to address WGS variant analysis in >5000 samples
Source: BMC Bioinformatics. 2016 Sep 10;17(1):361. doi: 10.1186/s12859-016-1211-6 (PMC5018196; doi:10.1186/s12859-016-1211-6)
Supplement: Additional file 1: — Figure S1. Venn Diagram of number of SNPs called by GATK-HC, GATK-UG, SNPTools and GotCloud and the FDR using HumanExome BeadChip array with 3533 shared samples as control. Figure S2. Rediscovery rate of SNP in the Exome region as function of alternate allele frequency using CHARGE WES variant call set as gold standard. The rediscovery rate exceeds 95% when alternate allele frequency f>=5x10^-4^ (AC>=5). Table S1. The choice of AWS instances used to deploy goSNAP for CHARGE WGS variant discovery in Stage A. All the jobs were scheduled via DNAnexus platform. Note that this list does not include the jobs for slicing and repacking in Stage A. Table S2. Instance specs in the “cost-effective” and “time-sensitive” mode of running goSNAP. Table S3. Profile of goSNAP runtime (in hour) with different region size, 100﻿ Kbp and 1 Mbp, and different instance specifications. Table S4. Profile of GATK-UG runtime (in hour) with different region size, 100 Kbp and 1 Mbp, and different instance specifications. (DOCX 232 kb) [file 12859_2016_1211_MOESM1_ESM.docx]

- **A hybrid computational strategy to address WGS variant analysis in >5000 samples**

Zhuoyi Huang*, Navin Rustagi*, Narayanan Veeraraghavan, Andrew Carroll, Richard Gibbs, Eric Boerwinkle, Manjunath Gorentla Venkata, Fuli Yu^§^

- *These authors have contributed equally to the paper
- § Please send questions about this document to fyu@bcm.edu
- **Comparison of multiple callers employed in goSNAP.**

Figure S1: Venn Diagram of number of SNPs called by GATK-HC, GATK-UG, SNPTools and GotCloud and the FDR using HumanExome BeadChip array with 3533 shared samples as control.

**Rare variant discovery rate of CHARGE WGS variant calling compared to CHARGE WES call set.**


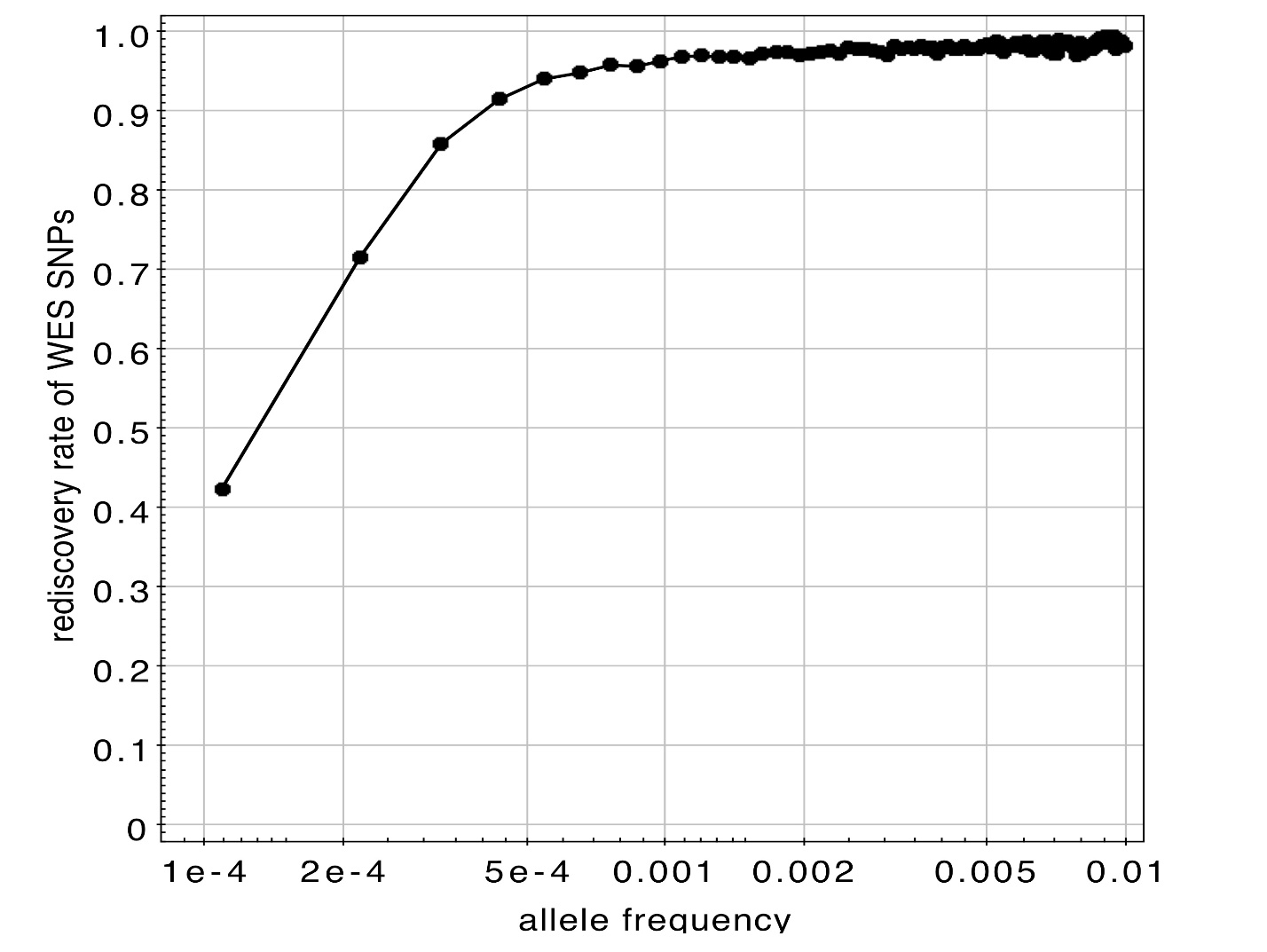


Figure S2: Rediscovery rate of SNP in the Exome region as function of alternate allele frequency using CHARGE WES variant call set as gold standard. The rediscovery rate exceeds 95% when alternate allele frequency f>=5x10^-4^ (AC>=5) .

**AWS instance types for CHARGE WGS variant calling jobs.**

| - Number of - processors | - Memory - (GB) | - Storage - (GB) | - Number of - 1Mbp regions |
| --- | --- | --- | --- |
| - 8 | - 61 | - 160 | - 934 |
| - 16 | - 122 | - 320 | - 606 |
| - 8 | - 15 | - 160 | - 595 |
| - 16 | - 30 | - 320 | - 506 |
| - 32 | - 60 | - 640 | - 57 |
| - 32 | - 244 | - 640 | - 24 |
| - 8 | 68.4 | - 1,680 | - 4 |
| - 32 | - 60.5 | - 3,360 | - 4 |

- Table S1. The choice of AWS instances used to deploy goSNAP for CHARGE WGS variant discovery in Stage A. All the jobs were scheduled via DNAnexus platform. Note that this list does not include the jobs for slicing and repacking in Stage A.

**Choice of instance types for goSNAP variant calling.**

The allocation of instance types for variant calling jobs is based on number of factors: 1) input alignment data size (in the sliced region), 2) expected runtime 3) possible failures. We defined two modes, “cost effective” and “time sensitive”, for goSNAP. If a job fails in the “cost effective” mode due to excess of memory requirements, or if fast turn-around time is desired, the job is executed in the “time sensitive” mode, which involves instances with higher memory specifications, and hence higher cost. The distributions of actually instance types used in CHARGE WGS calling is given in TABLE S1.

| Input BAM  tarball size (GB) | Instance specs in “cost-effective” mode | Instance specs in “time-sensitive” mode |
| --- | --- | --- |
| >1680 | MEM=60.5GB,  HDD=3360GB,  Cores=32 | MEM=60.5GB,  HDD=3360GB,  Cores=32 |
| 640-1680 | MEM=68.4GB,  HDD=1680GB,  Cores=8 | MEM=68.4GB,  HDD=1680GB,  Cores=8 |
| 320-640 | MEM=60GB,  SSD=640GB,  Cores=32 | MEM=244GB,  SSD=640GB,  Cores=32 |
| 160-320 | MEM=30GB,  SSD=320GB,  Cores=16 | MEM=122GB,  SSD=320GB,  Cores=16 |
| <160 | MEM=15GB,  SSD=160GB,  Cores=8 | MEM=61.0GB,  SSD=160GB,  Cores=8 |

Table S2: Instance specs in the “cost-effective” and “time-sensitive” mode of running goSNAP.

**goSNAP runtime profiling using different region size and instance types.**

In Stage A, goSNAP pipeline was profiled on two window sizes, 100Kbp and 1Mbp, and a number of combinations of instance specs. Profiling for larger window sizes was not performed due to limits on instance storage sizes and runtime limit. We profiled the runtime of goSNAP with all four callers (Table S3) and GATK-UnifiedGenotyper (GATK-UG) alone (Table S4). In each case, we evaluated the runtime for the full run in the entire region, with retries on GATK-UG errors [1] until the calling was successful (denoted “with GATK-UG error”), and the runtime subtracted the time used in retries on error (denoted “if without GATK-UG error”). As the GATK-UG “somehow” error has a sporadic manner and related to memory allocation, the error rate is lower with the smaller region size. However, the runtime does not scale linearly with the region size. As a result, the choice of region size reflects the tradeoff between effective runtime and error rate.

| region size  100Kbp | with GATK-UG error | | if without GATK-UG error | |
| --- | --- | --- | --- | --- |
|  | #core=8 | #core=32 | #core=8 | #core=32 |
| mem=16GB | 9.83 |  | 9.83 |  |
| mem=64GB | 10.78 | 4.12 | 10.78 | 4.12 |
| mem=256GB |  | 4.40 |  | 4.40 |
| region size  1Mbp | with GATK-UG error | | if without GATK-UG error | |
|  | #core=8 | #core=32 | #core=8 | #core=32 |
| mem=16GB | 60.25 |  | 44.97 |  |
| mem=64GB | 60.05 | 22.05 | 47.02 | 20.09 |
| mem=256GB |  | 25.22 |  | 21.58 |

Table S3: Profile of goSNAP runtime (in hour) with different region size, 100Kbp and 1Mbp, and different instance specifications.

| region size  100Kbp | with GATK-UG error | | if without GATK-UG error | |
| --- | --- | --- | --- | --- |
|  | #core=8 | #core=32 | #core=8 | #core=32 |
| mem=16GB | 1.14 |  | 1.14 |  |
| mem=64GB | 1.35 | 0.66 | 1.35 | 0.66 |
| mem=256GB |  | 0.73 |  | 0.73 |
| region size  1Mbp | with GATK-UG error | | if without GATK-UG error | |
|  | #core=8 | #core=32 | #core=8 | #core=32 |
| mem=16GB | 24.10 |  | 13.16 |  |
| mem=64GB | 22.42 | 9.98 | 13.08 | 6.63 |
| mem=256GB |  | 9.89 |  | 7.04 |

Table S4: Profile of GATK-UG runtime (in hour) with different region size, 100Kbp and 1Mbp, and different instance specifications.

- [1] UnifiedGenotyper error: “*Somehow the requested coordinate is not covered by the read.*” <http://gatkforums.broadinstitute.org/discussion/3141/unifiedgenotyper-error-somehow-the-requested-coordinate-is-not-covered-by-the-read>. Accessed 25^th^ October 2015.
